# Age differences in the relationships between risk factors and loss of kidney function: a general population cohort study

**DOI:** 10.1186/s12882-020-02121-z

**Published:** 2020-11-13

**Authors:** Tadashi Toyama, Kiyoki Kitagawa, Megumi Oshima, Shinji Kitajima, Akinori Hara, Yasunori Iwata, Norihiko Sakai, Miho Shimizu, Atsushi Hashiba, Kengo Furuichi, Takashi Wada

**Affiliations:** 1 Department of Nephrology and Laboratory Medicine, Kanazawa University, Kanazawa, Japan; 2 Innovative Clinical Research Center, Kanazawa University, Kanazawa, Japan; 3 Division of Internal Medicine, National Hospital Organization Kanazawa Medical Center, Kanazawa, Japan; 4 Kanazawa Medical Association, Kanazawa, Japan; 5 Department of Nephrology, Kanazawa Medical University School of Medicine, Uchinada, Japan

**Keywords:** Aged, Chronic renal insufficiency, Glomerular filtration rate

## Abstract

**Background:**

Annual decline in kidney function is a widely applied surrogate outcome of renal failure. It is important to understand the relationships between known risk factors and the annual decline in estimated glomerular filtration rate (eGFR) according to baseline age; however, these remain unclear.

**Methods:**

A community-based retrospective cohort study of adults who underwent annual medical examinations between 1999 and 2013 was conducted. The participants were stratified into different age groups (40–49, 50–59, 60–69, 70–79, and ≥ 80 years) to assess the risk for loss of kidney function. A mixed-effects model was used to estimate the association between risk factors and annual changes in eGFR.

**Results:**

In total, 51,938 participants were included in the analysis. The age group of ≥80 years included 8127 individuals. The mean annual change in eGFR was − 0.39 (95% confidence interval: − 0.41 to − 0.37) mL/min/1.73 m^2^ per year. Older age was related to faster loss of kidney function. In the older age group, higher systolic blood pressure, proteinuria, and current smoking were related to faster loss of kidney function (*p* trend < 0.01, 0.03, and < 0.01, respectively). Conversely, each age group showed similar annual loss of kidney function related to lower hemoglobin levels and diabetes mellitus (*p* trend 0.47 and 0.17, respectively).

**Conclusions:**

Higher systolic blood pressure, proteinuria, and smoking were related to faster loss of kidney function, and a greater effect size was observed in the older participants. More risk assessments for older people are required for personalized care.

**Supplementary Information:**

The online version contains supplementary material available at 10.1186/s12882-020-02121-z.

## Background

Chronic kidney disease (CKD) is a risk factor for cardiovascular and all-cause death, not only in middle-aged people but also in older people [1–4]. Studies have revealed related risk factors, including hypertension, diabetes mellitus, or tobacco use, for the development of CKD [5, 6].

Older people, a major population in the efforts to prevent CKD and end-stage renal disease (ESKD), have a different physiology from young and middle-aged populations, which should be considered in the treatment plan. However, knowledge of the risk factors for kidney dysfunction, particularly in older people, is sparse.

A decline in kidney function occurs with the natural physiological course of aging [7]. CKD is a common condition in older people, as nearly half of the population aged ≥80 years have an estimated glomerular filtration rate (eGFR) of ≤60 mL/min/1.73 m^2^ [8]. Treatment of older people requires an integrated plan that considers these associated risks to provide them with appropriate care [7]. The increased incidence of ESKD is becoming a serious problem worldwide [9]. For example, > 28,000 people aged ≥75 years in the United States have ESKD [10]. To provide more personalized treatment for preventing age-related kidney disease, basic information about risk factors for age-associated loss of kidney function is important.

Therefore, we conducted a cohort study in the general population to examine age differences in the relationship between the comprehensively known risk factors and loss of kidney function.

## Methods

### Study participants

This was a community-based historical cohort study of adults who underwent annual medical examinations from 1999 to 2013 in Kanazawa City, Ishikawa Prefecture, Japan. All adults aged ≥40 years were eligible to undergo medical examinations. Individuals whose serum creatinine levels were measured and who were followed up at least once during the observation period were included in the analysis. There was no upper limit of age for inclusion criteria. Individuals were excluded from the analysis if they refused to participate, lacked baseline data of covariates, or lacked follow-up eGFR data.

### Measurement of risk factors

Baseline clinical parameters, including, age, sex, blood pressure, treatment of hypertension, body mass index (BMI), urinary protein, hemoglobin, total cholesterol, smoking status, history of coronary disease, and history of stroke, were recorded. Blood pressure was measured in the sitting position after a rest period. Random spot urine samples were assessed by urinary dipstick test strips, and the results were classified as negative/trace or ≥ 1+ (1+ corresponds to approximately 30 mg/dL of urine protein). eGFR was calculated from serum creatinine levels measured by enzymatic methods using the equation for Japanese people [11]. In 1999–2001, creatinine was measured using the Jaffe’s reaction. From 2002, it was measured using enzymatic methods. Serum creatinine measured by the Jaffe’s reaction was calibrated by subtracting 0.2 mg/dL [12]. Diabetes mellitus was defined by the following criteria: glycated hemoglobin ≥6.5%, fasting plasma glucose ≥126 mg/dL (≥7.0 mmol/L), or treatment of diabetes mellitus [13]. Information on current smoking status (regardless of smoking history), history of coronary artery disease (angina or myocardial infarction), and history of stroke (hemorrhagic or ischemic stroke) was obtained from questionnaires.

### Statistical analysis

The participants were categorized into five groups according to age (40–49, 50–59, 60–69, 70–79, and ≥ 80 years) at baseline. Analyses were conducted according to the age-stratified groups. Continuous variables are presented as mean and standard deviation, whereas categorical variables are presented as proportions. Linear trends in baseline characteristics were tested between the age categories using linear or logistic regression analysis, appropriately.

The outcome of this study was the eGFR slope represented by annual changes in eGFR (mL/min/1.73 m^2^ per year) during the follow-up period. To estimate annual changes in eGFR in each individual, a mixed-effects model with a random intercept and a random slope was applied [14, 15]. Covariates used in adjustment were selected according to previous reports on risk factors for kidney dysfunction [5, 16–19]. Baseline risk factors (covariates) included: age, sex, systolic blood pressure, diastolic blood pressure, proteinuria, hemoglobin, total cholesterol, current smoking status, history of coronary disease, history of stroke, and diabetes mellitus. Similarly, covariate-time interaction terms were used as covariates. To consider underlying risks related to blood pressure, values of 10 and 5 mmHg were constantly added to systolic and diastolic blood pressure, respectively, in participants treated for hypertension [20, 21]. Adjusted means and 95% confidence intervals (CIs) for annual changes in eGFR were estimated. Weighted linear regression analysis with inverse variance weighting was used to test linear trends between the mean ages of each age group and the estimated annual changes in eGFR [22].

The two-tailed significance level was set at *p* < 0.05, and all analyses were performed using Stata/MP statistical software (version 14.2; StataCorp LP, College Station, TX, USA).

## Results

### Selection of participants and baseline characteristics

Of 133,925 individuals who underwent a medical examination in Kanazawa City between 1999 and 2013, 51,938 met the eligibility criteria and were included in the analysis (Supplementary Figure 1). The median follow-up period was 4.0 years.

Table 1 shows the participants’ baseline characteristics. The group with the highest number of participants was the 60–69 years age group (*n* = 19,015). Lower eGFR and higher blood pressure were observed in the older group, and more than half of the participants aged ≥70 years were treated for hypertension. Nearly half of the participants (49%) in the age group of ≥80 years had an eGFR of < 60 mL/min/1.73 m^2^. All groups showed quite similar levels of hemoglobin, BMI, and total cholesterol. Diabetes mellitus, history of coronary artery disease, and history of stroke were more frequent in older people than in younger people. The proportion of current smokers was the highest in the youngest group (26%).

**Table 1:** Baseline characteristics of study population

			Age (years)				
Variable	40–49 (*n* = 1775)	50–59 (*n* = 4386)	60–69 (*n* = 19,015)	70–79 (*n* = 18,635)	80 – (*n* = 8127)	Overall (*n* = 51,938)	*p* trend
Age (years)	45 ± 3	57 ± 3	65 ± 3	75 ± 3	85 ± 4	70 ± 10	< 0.01
Sex (men)	34%	32%	39%	39%	33%	37%	0.71
eGFR (mL/min/1.73 m^2^)	86 ± 15	79 ± 14	75 ± 14	69 ± 15	61 ± 17	72 ± 16	< 0.01
eGFR < 60 mL/min/1.73 m^2^	2%	6%	12%	25%	49%	22%	< 0.01
eGFR < 60 mL/min/1.73 m^2^ and/or proteinuria (≥1+)	7%	11%	17%	29%	53%	26%	< 0.01
Systolic blood pressure (mmHg)	118 ± 17	127 ± 18	132 ± 18	136 ± 17	139 ± 18	134 ± 18	< 0.01
Diastolic blood pressure (mmHg)	73 ± 12	78 ± 12	79 ± 11	77 ± 10	75 ± 11	77 ± 11	< 0.01
Hemoglobin (g/dL)	13.5 ± 1.7	13.7 ± 1.4	13.7 ± 1.3	13.2 ± 1.4	12.4 ± 1.4	13.3 ± 1.4	< 0.01
Body mass index (kg/m^2^)	22.3 ± 3.7	22.7 ± 3.4	22.9 ± 3.1	23.2 ± 3.2	22.5 ± 3.4	22.9 ± 3.3	0.01
Total cholesterol (mg/dL)	201 ± 34	214 ± 36	209 ± 34	201 ± 33	195 ± 33	204 ± 34	< 0.01
Proteinuria (≥1+)	5%	5%	6%	7%	11%	7%	< 0.01
Diabetes mellitus	2%	7%	10%	13%	12%	11%	< 0.01
Current smoker	26%	19%	14%	10%	5%	12%	< 0.01
Treatment of hypertension (%)	7%	22%	36%	51%	60%	43%	< 0.01
History of coronary artery disease (%)	2%	4%	9%	16%	26%	13%	< 0.01
History of stroke (%)	1%	3%	5%	9%	13%	7%	< 0.01
Follow-up period (years)	2.8 (1.1, 4.0)	3.2 (1.8, 4.9)	4.0 (2.1, 4.9)	4.2 (2.8, 5.0)	3.3 (2.0, 4.9)	4.0 (2.1, 4.9)	< 0.01
Number of creatinine measurements (n)	3 (2, 4)	3 (2, 5)	4 (3, 6)	5 (3, 6)	4 (2, 5)	4 (3, 6)	< 0.01

Continuous variables are expressed as mean ± standard deviation, or median (25th and 75th percentiles). Categorical variables are expressed as numbers (percentage)

Abbreviation: *eGFR* estimated glomerular filtration rate

### Slope of eGFR and relationships between risk factors

The adjusted mean eGFR slope in all participants was − 0.39 mL/min/1.73 m^2^ per year. Significant declines in kidney function were observed in the ≥60 years age groups (Table 2). Most of the known risk factors were associated with a significantly faster decline in eGFR, except for higher total cholesterol, which was related to a slower decline in eGFR (Table 3). Proteinuria, current smoking, and diabetes mellitus were associated with a greater decline in eGFR (− 0.58 [95% CI: − 0.67, − 49], − 0.25 [95% CI: − 0.32, − 0.18], and − 0.33 [95% CI: − 0.40, − 0.26] mL/min/1.73 m^2^ per year, respectively). Similarly, Higher systolic blood pressure (+ 10 mmHg) and lower hemoglobin (− 1 g/dL) were associated with a greater decline in eGFR (− 0.16 [95% CI: − 0.17, − 0.14] and − 0.16 [95% CI: − 0.18, − 0.15] mL/min/1.73 m^2^ per year, respectively).

**Table 2:** Adjusted mean slope according to age groups

Age (years)		Adjusted mean slope^a^ (mL/min/1.73 m^2^ per year)		*p*-value
All participants	(*n* = 51,938)	−0.39	(− 0.41, − 0.37)	< 0.01
40–49	(*n* = 1775)	0.02	(− 0.12, 0.16)	0.82
50–59	(*n* = 4386)	− 0.01	(− 0.08, 0.07)	0.86
60–69	(*n* = 19,015)	− 0.16	(− 0.19, − 0.13)	< 0.01
70–79	(*n* = 18,635)	− 0.53	(− 0.56, − 0.49)	< 0.01
≥80	(*n* = 8127)	−0.87	(− 0.94, − 0.80)	< 0.01

Data are presented as mean and 95% confidence interval

^a^Adjusted for sex, systolic blood pressure, diastolic blood pressure, body mass index, proteinuria, hemoglobin, total cholesterol, smoking status, history of coronary disease, history of stroke, and diabetes mellitus

**Table 3:** Differences from mean slope according to risk factors

Variables	Difference from mean slope^a^ (mL/min/1.73 m^2^ per year)		*p*-value
Men (vs. Women)	−0.18	(− 0.24, − 0.13)	< 0.01
Systolic blood pressure (+ 10 mmHg)	−0.16	(− 0.17, − 0.14)	< 0.01
Diastolic blood pressure (+ 5 mmHg)	0.04	(0.02, 0.05)	< 0.01
Body mass index (+ 1)	− 0.02	(− 0.02, − 0.01)	< 0.01
Proteinuria 1+ (vs. normal/trace)	− 0.58	(− 0.67, − 0.49)	< 0.01
Hemoglobin (−1 g/dL)	−0.16	(− 0.18, − 0.15)	< 0.01
Total cholesterol (+ 10 mg/dL)	0.02	(0.01, 0.03)	< 0.01
Current smoking (vs. no current smoking)	− 0.25	(− 0.32, − 0.18)	< 0.01
History of coronary disease (vs. no history)	− 0.15	(− 0.21, − 0.08)	< 0.01
History of stroke (vs. no history)	−0.10	(− 0.19, − 0.01)	0.02
Diabetes mellitus (vs. no diabetes)	− 0.33	(− 0.40, − 0.26)	< 0.01

*n* = 51,938. Data are presented as mean and 95% confidence interval

^a^Values are differences from the adjusted mean slope of all participants (*n* = 51,938). Each variable was adjusted for all other variables

### Slope of eGFR according to age groups

**Fig. 1:**
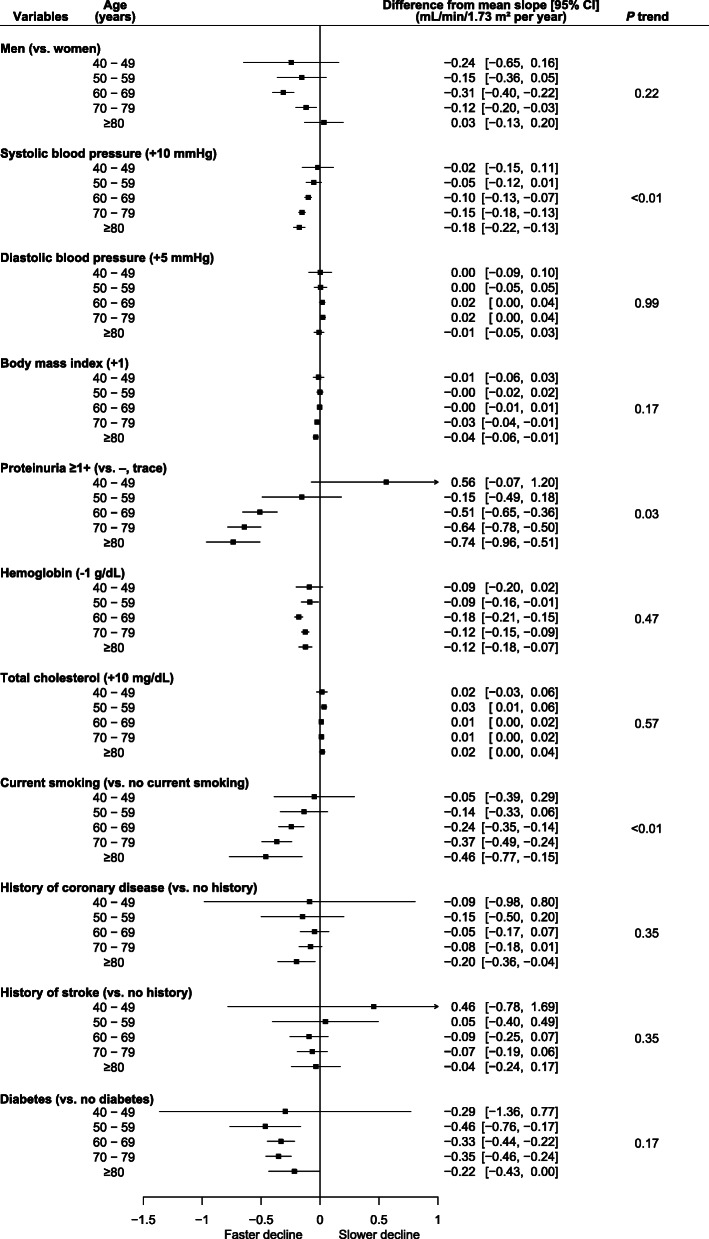
Risk factors for loss of kidney function and differences from the mean estimated glomerular filtration rate slopes according to age. Number of participants in each group: *n* = 1775 (age 40–49 years); *n* = 4396 (age 50–59 years); *n* = 19,015 (age 60–69 years); *n* = 18,635 (age 70–79 years); and *n* = 8127 (age ≥ 80 years). Values represent differences from the mean slopes of each age group. Each variable was adjusted for all other variables. The error bars represent 95% confidence intervals. The *p* trend value was obtained to test the consistency of the age relationships among the age groups

The differences in eGFR decline according to the age group for each risk factor are shown in Fig. 1. Higher systolic blood pressure, proteinuria, and current smoking status were related to a faster decline in eGFR in the older age groups (*p* trend < 0.01, 0.03, and < 0.01, respectively). Lower hemoglobin and diabetes mellitus status were related to a significantly greater eGFR decline in each age group, except for the youngest age group (age 40–49 years), and no significant trends, according to the age group, were observed (*p* trend 0.47 and 0.17, respectively).

**Fig. 2:**
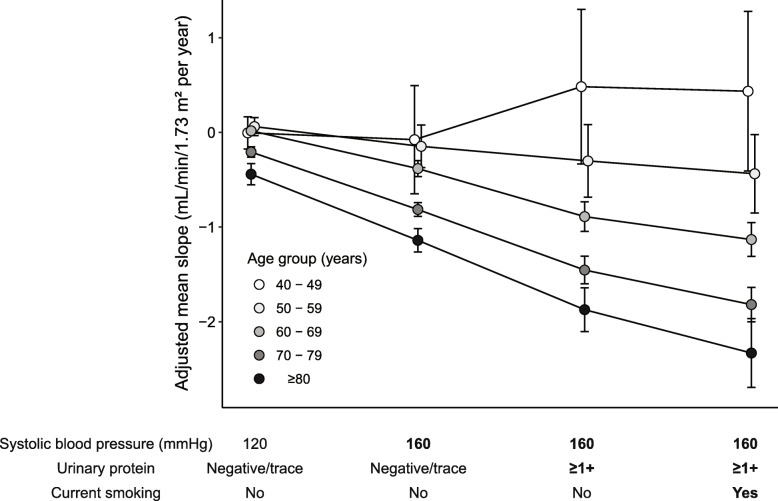
Estimated glomerular filtration rate (eGFR) slopes according to age groups with respect to representative risk factors. Values are mean (95% confidence intervals) changes in eGFR slopes adjusted for sex, systolic blood pressure, diastolic blood pressure, body mass index, proteinuria, hemoglobin, total cholesterol, smoking status, history of coronary disease, history of stroke, and diabetes mellitus. For variables not mentioned in the axis labels, the average values were applied for each age group. The values of the representative risk factors (blood pressure, urinary protein, current smoking status) were chosen arbitrarily

The representative estimated eGFR slopes according to age groups and risk factors (hypertension, proteinuria, and smoking status) are shown in Fig. 2. The participants without any risks (systolic blood pressure 120 mmHg, negative or trace urinary protein, and without current smoking) had almost the same mean eGFR slopes among the age groups. In a stepwise analysis of additional risks, more differences in eGFR slope according to age groups were observed.

For further trend analysis, systolic blood pressure was classified into seven categories (< 110, 110–119, 120–129, 130–139, 140–149, 150–159, and ≥ 160 mmHg). Higher systolic blood pressure was associated with an evidently faster decline in eGFR in the ≥60 years age groups. U-shaped associations were not observed in any age group (Supplementary Figure 2).

Similar results were obtained in sensitivity analyses using systolic and diastolic blood pressure without constant addition according to the treatment status at the time of measurement (Supplementary Figure 3) and in analyses (combining participants in the 40–49 years and 50–59 years age groups) (Supplementary Figure 4).

## Discussion

In this community-based longitudinal study of adults, the risks related to a faster decline in eGFR were different among age groups. In relation to hypertension, proteinuria, and smoking status, faster declines in eGFR were observed in the older group. Other risk factors, including anemia and diabetes mellitus, were related to a similar rate of eGFR decline among the age groups.

Age is one of the most fundamental considerations for providing personalized care. The risk factors for kidney dysfunction have been evaluated in cohort studies or interventional trials [5, 23]. For example, a study of a general Japanese population showed that the relationship between blood pressure and GFR slopes varied across age groups [24]. However, age-adjusted risks, rather than age-stratified risks, have usually been evaluated, and thus, reported risk factors are limited. To achieve more personalized care, we conducted an age-stratified risk assessment of comprehensive risk factors for loss of kidney function.

### Clinical significance and study implications

In this study, higher systolic blood pressure was related to a faster loss of kidney function in older people. This result was consistent with those of previous studies reporting that the absence of hypertension was related to a slower progression of kidney disease in older people [24, 25]. A possible mechanism underlying this association might be glomerular hypertension by hyalinization of afferent arterioles [26] that may affect the autoregulation of glomerular blood flow [27]. Because active treatment of hypertension is a risk factor for kidney failure [28], a study of renal outcome regarding hypertension treatment in older people is required to evaluate the balanced target of blood pressure treatment in older people.

Current smoking is a known risk factor for the loss of kidney function in middle-aged or older people [29]. Interestingly, this relationship with smoking was observed, even in the participants aged ≥80 years. Current smoking was not associated with reduced kidney function in young people; however, this result should be carefully interpreted because smoking may transiently increase eGFR [30]. In addition, the elevated eGFR observed in individuals with proteinuria aged 40 to 49 years should be interpreted with caution because it may be related to single-nephron hyperfiltration [31], which could result in kidney dysfunction.

In this study, higher BMI was related to a slightly faster loss of kidney function in older people. However, no age-dependent trend was observed. Moreover, the effect sizes related to higher BMI were minimal in each age group. Considering that most participants had BMI < 30 kg/m^2^, this result was consistent with those of other studies that reported that a BMI of 25 to 30 kg/m^2^ was not related to a faster loss of kidney function in any age group [32].

Interestingly, a history of stroke or coronary disease did not relate to the loss of kidney function in younger people. A study by Esposito et al. reported a faster loss of kidney function in younger people with CKD than in the elderly [33]. Considering the differences in the study design, the stable kidney function observed in younger people in our study may be because many of them had better kidney function (only 2% had an eGFR of < 60 ml/min/1.73 m^2^) and fewer complications of hypertension (7%) or diabetes (2%). Similarly, it may be because of the strict medical treatments after the event of stroke or coronary disease. In contrast, the rate of decline in kidney function in older people did not differ significantly from that in previous studies. For example, a US study reported annual rates of eGFR decline of 0.8 and 1.4 mL/min/1.73 m^2^ in non-diabetic men and women aged 66 years and older, respectively [23].

Due to the large sample size, this study might have detected a minimal difference in the GFR slope. However, most of the significantly different risk factors observed in this study had a difference of − 0.10 mL/min/1.73 m^2^ from the mean slope. Considering that the mean slope was − 0.39, the presence of multiple factors could lead to a clinically significant loss of kidney function. Furthermore, risk factors that increased in association with older age might become more important over time, which is shown in Fig. 2.

### Strengths and limitations

More than 50,000 Japanese people aged 40 to 80 years or older participated in this study. The participants may not be different from the typical Japanese population. The mean eGFR slope in the study population was − 0.39 mL/min/1.73 m^2^ per year, which was almost the same as that reported in a nationwide study in Japan [24]. Furthermore, the wide range of characteristics may contribute to the generalizability of the results.

Another strength of this study was the inclusion of > 8000 participants aged ≥80 years. Old-age populations are usually not stratified because of either their limited number or they are not the target population. The population of people aged ≥80 years is increasing worldwide [34]. The results of this study provide basic information about the risk factors of loss of kidney function in older people. Interventional trials, including those comprising people of this age group, are warranted to validate the results of this study.

This study has several limitations. First, drug classes for the treatment of hypertension or diabetes mellitus were not considered in the analysis. Some medications, including angiotensin II receptor blockers or sodium-glucose cotransporter-2 inhibitors, may affect kidney function [35]. Second, we did not investigate the underlying diseases of kidney dysfunction that might affect the progression of the disease. Possibly, primary diseases that were not examined in this study, such as polycystic kidney disease, might have had a significant impact on renal function [36]. Furthermore, the high prevalence of kidney dysfunction and proteinuria in the elderly might indicate that the effects of disease-related treatments could not be fully adjusted. Third, smoking history was not included in the questionnaire. Because the questionnaire asked about the current smoking status at the time of examination, the risks related to smoking in older people may have been underestimated. Fourth, previous studies of kidney function have observed that annual changes in eGFR over a 3-year follow-up period can be used as a surrogate outcome for assessing the risk of ESKD, even in those with preserved kidney function [37, 38]; however, the short median follow-up period of 2.8 years in the youngest age group may affect the ability to detect changes in eGFR. Fifth, this study was not a cluster-randomized observational study, and the limited number of participants, especially those in their 40s, might have led to a selection bias toward those with health problems. Sixth, kidney dysfunction and proteinuria observed in the elderly might have been associated with all-cause and cardiovascular mortality [39], which could cause a bias towards attenuating a decline in kidney function.

## Conclusion

In the general population, higher systolic blood pressure, proteinuria, and smoking status were related to greater loss of kidney function in older people. The differences in risks according to age should be considered, and interventional studies dedicated to each age group are needed to clarify the causal relationships.

## Supplementary Information

**Additional file 1: Figure S1.** Flow diagram of the selection of study participants. **Figure S2.** Baseline systolic blood pressure and loss of kidney function according to age groups. **Figure S3.** Risk factors for loss of kidney function and differences from the mean estimated glomerular filtration rate slopes according to age (analysis using unadjusted systolic and diastolic blood pressure). **Figure S4.** Risk factors for loss of kidney function and differences from the mean estimated glomerular filtration rate slopes according to age (analysis of age 40–49 years merged with 50–59 years).

## Data Availability

The data supporting our study findings are available from Kanazawa Medical Association, but restrictions apply to the availability of these data, which were used under license for the current study, and so are not publicly available. Data are, however, available from the authors upon reasonable request and with permission of Kanazawa Medical Association.

## Abbreviations

eGFR: Estimated glomerular filtration rate
CKD: Chronic kidney disease
ESKD: End-stage renal disease
BMI: Body mass index
CI: Confidence interval
