# Supplementary material for: Age differences in the relationships between risk factors and loss of kidney function: a general population cohort study
Source: BMC Nephrol. 2020 Nov 13;21:477. doi: 10.1186/s12882-020-02121-z (PMC7664087; doi:10.1186/s12882-020-02121-z)
Supplement: Supplementary file 1 — Additional file 1: Figure S1. Flow diagram of the selection of study participants. Figure S2. Baseline systolic blood pressure and loss of kidney function according to age groups. Figure S3. Risk factors for loss of kidney function and differences from the mean estimated glomerular filtration rate slopes according to age (analysis using unadjusted systolic and diastolic blood pressure). Figure S4. Risk factors for loss of kidney function and differences from the mean estimated glomerular filtration rate slopes according to age (analysis of age 40–49 years merged with 50–59 years). [file 12882_2020_2121_MOESM1_ESM.docx]

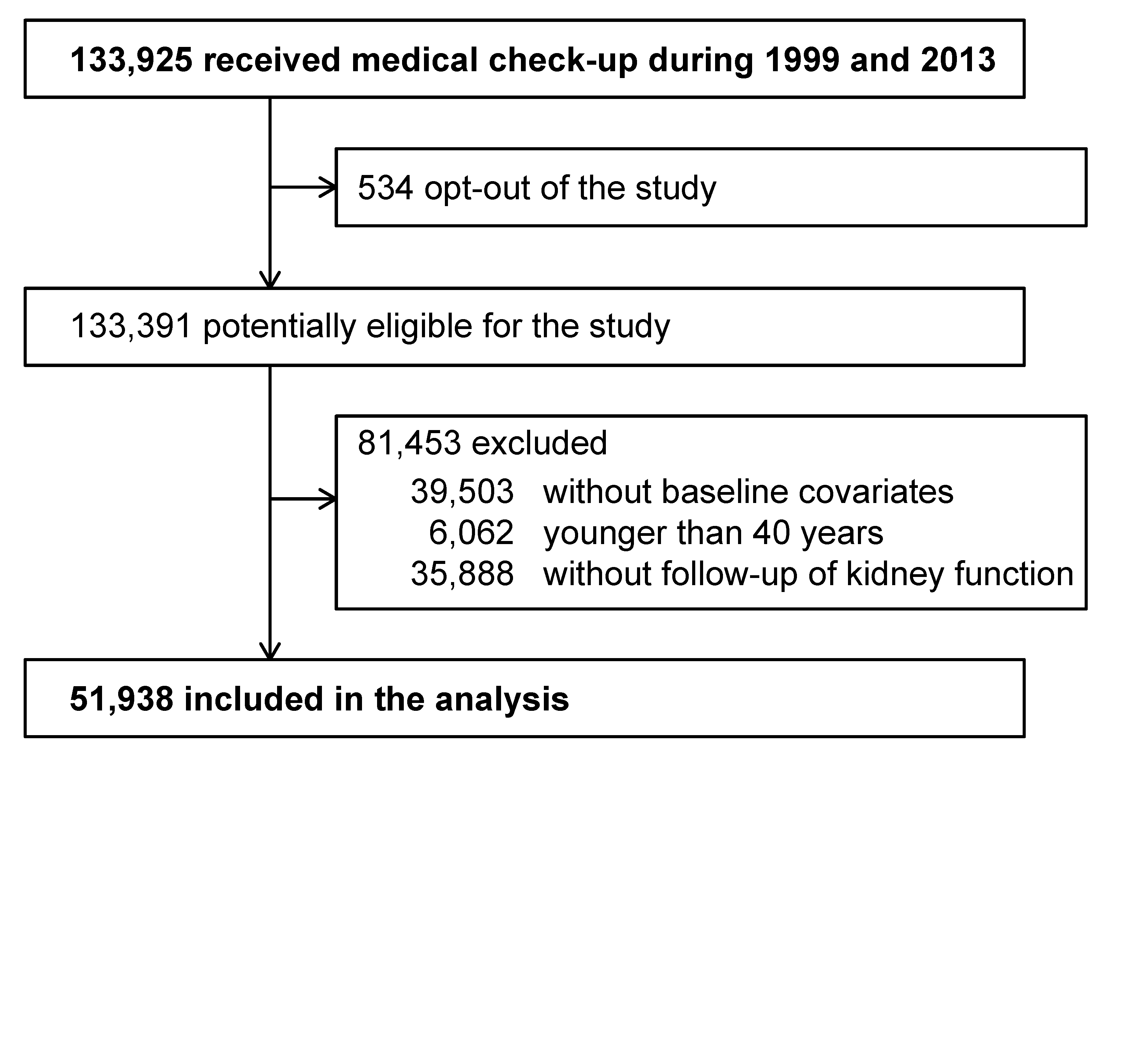


**Supplementary Figure 1.** Flow diagram of the selection of study participants.


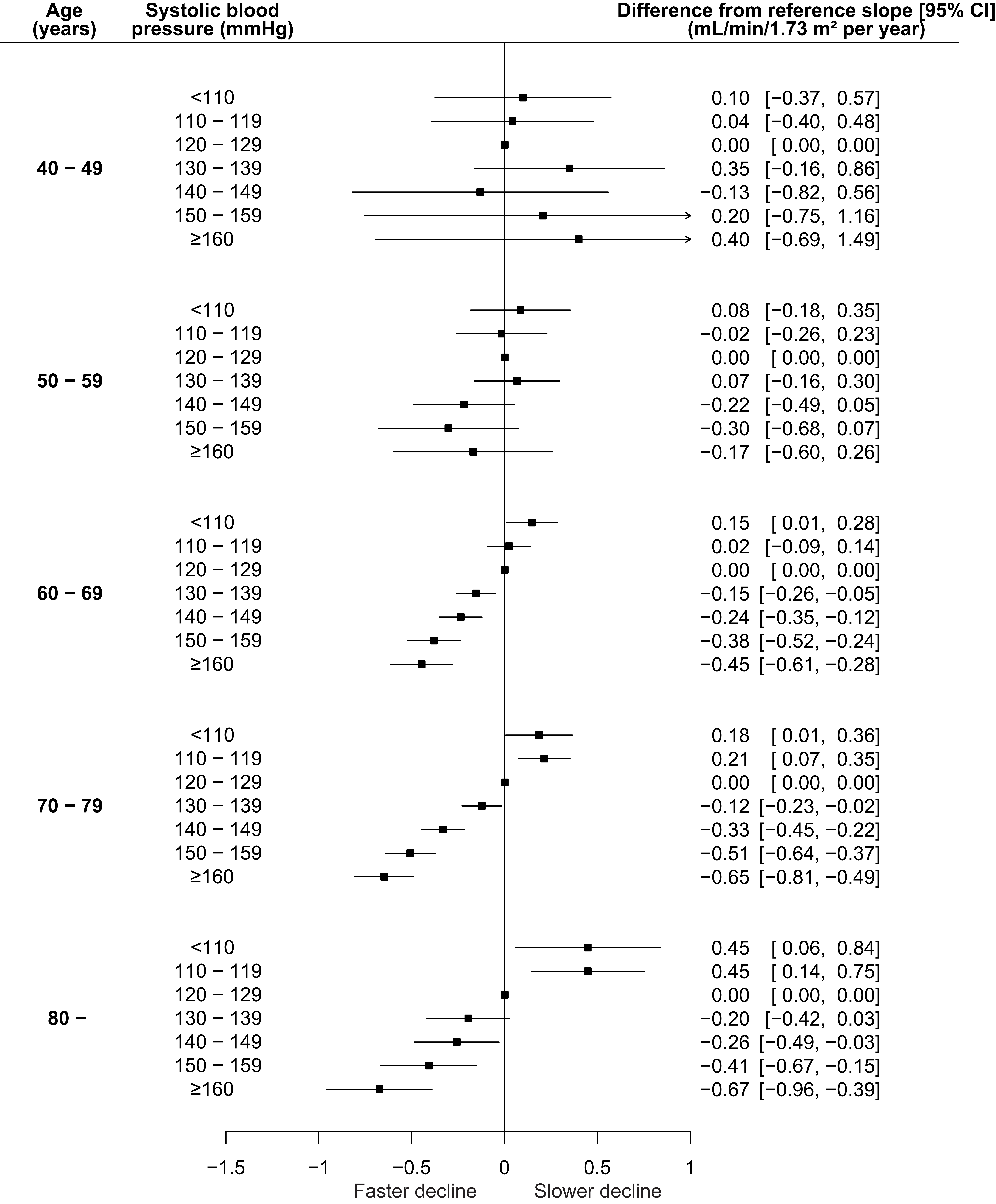


**Supplementary Figure 2.** Baseline systolic blood pressure and loss of kidney function according to age groups.

Systolic blood pressure was divided at 110, 120, 130, 140, 150, 160 mmHg.

Number of people in each group: n=1,775 (age 40–49 years); n=4,396 (age 50–59 years); n=19,015 (age 60–69 years); n=18,635 (age 70–79 years); n=8,127 (age ≥80 years). Values are differences from the slope of reference group (systolic blood pressure 120–129 mmHg). Each variable was adjusted for sex, diastolic blood pressure, body mass index, proteinuria, hemoglobin, total cholesterol, smoking status, history of coronary disease, history of stroke, and diabetes mellitus. The error bars represent 95% confidence intervals.


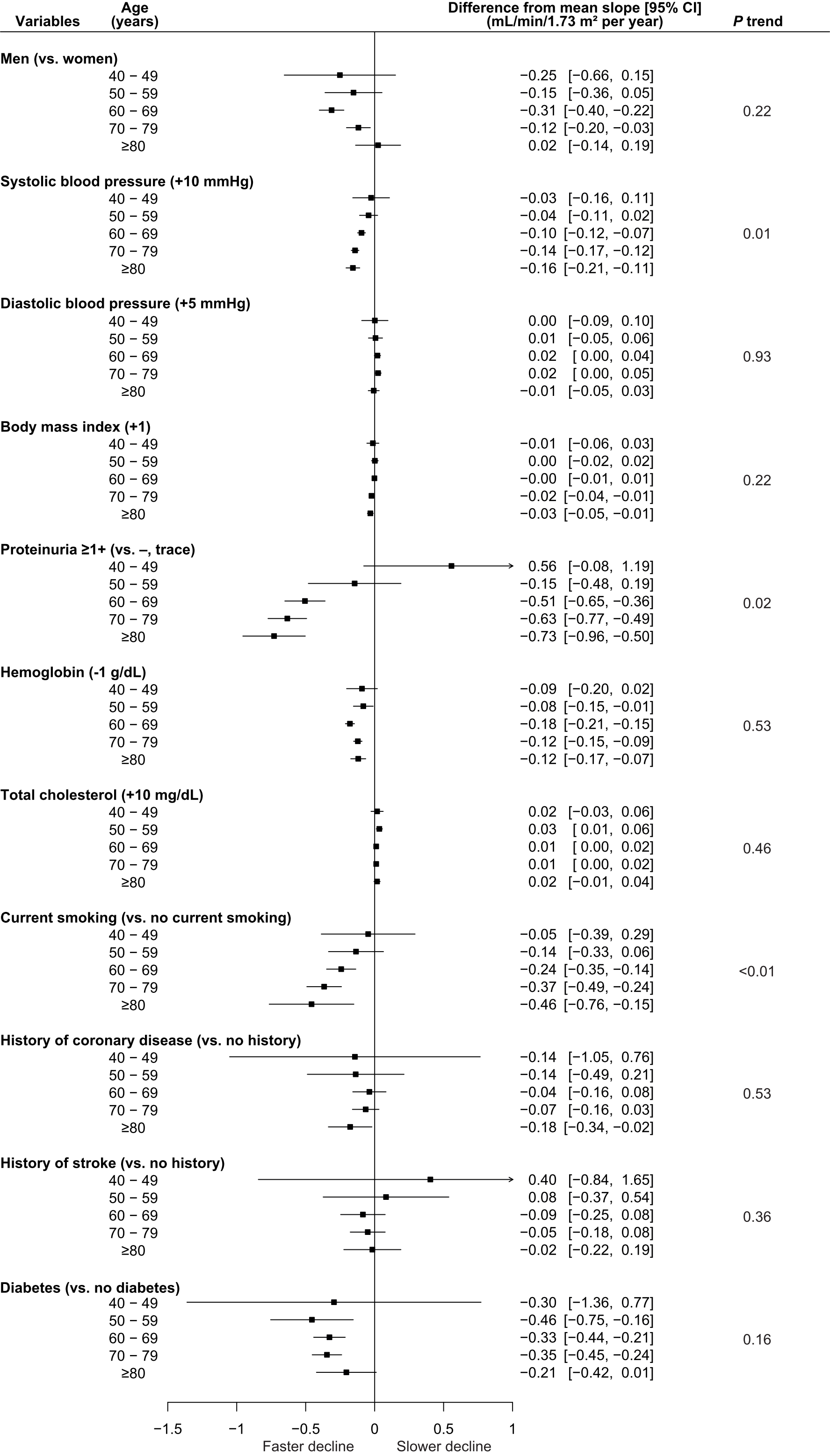


**Supplementary Figure 3.** Risk factors for loss of kidney function and differences from the mean estimated glomerular filtration rate slopes according to age (analysis using unadjusted systolic and diastolic blood pressure)

Number of participants in each group: n=1,775 (age 40–49 years); n=4,396 (age 50–59 years); n=19,015 (age 60–69 years); n=18,635 (age 70–79 years); and n=8,127 (age ≥80 years). Values represent differences from the mean slopes of each age group. Each variable was adjusted for all other variables. The error bars represent 95% confidence intervals. The *p* trend value was obtained to test the consistency of the age relationships among the age groups. Systolic and diastolic blood pressures were used without adjustment for treatment status at the time of measurement, and treatment of hypertension was added to the covariates.


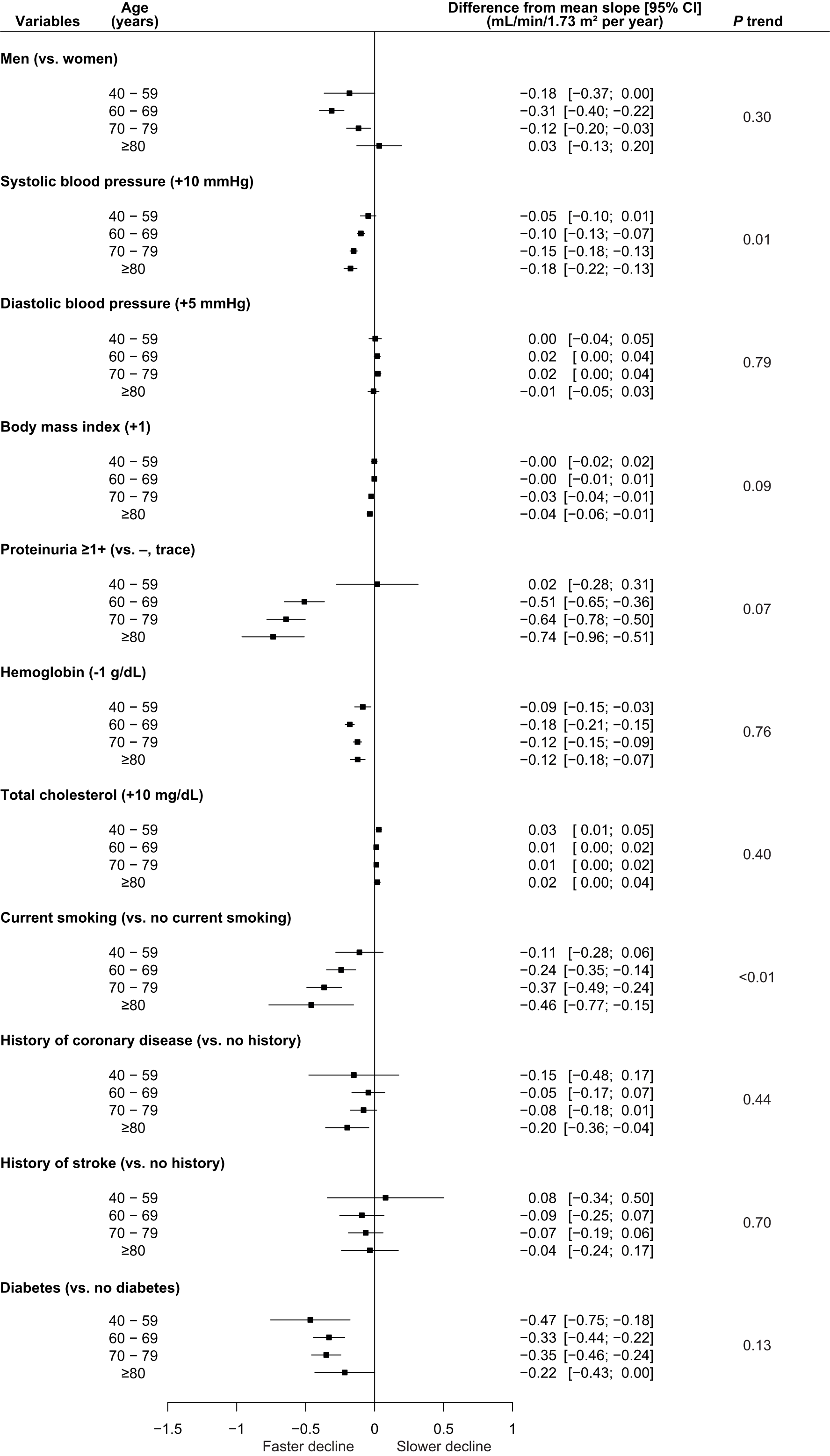


**Supplementary Figure 4.** Risk factors for loss of kidney function and differences from the mean estimated glomerular filtration rate slopes according to age (analysis of age 40–49 years merged with 50–59 years)

Number of participants in each group: n=6,171 (age 40–59 years); n=19,015 (age 60–69 years); n=18,635 (age 70–79 years); and n=8,127 (age ≥80 years). Values represent differences from the mean slopes of each age group. Each variable was adjusted for all other variables. The error bars represent 95% confidence intervals. The *p* trend value was obtained to test the consistency of the age relationships among the age groups.
